# The Influence of Different Anatomical Sites on the Characteristics and Differentiation Capacity of Rabbit Adipose‐Derived Mesenchymal Stem Cells

**DOI:** 10.1155/vmi/7537562

**Published:** 2026-05-21

**Authors:** Suryo Kuncorojakti, Ahmad Aswin, Diyantoro, Helen Susilowati, Ira Sari Yudaniayanti, Yeni Dhamayanti, Lina Susanti, Watchareewan Rodprasert

**Affiliations:** ^1^ Department of Veterinary Science, Faculty of Veterinary Medicine, Universitas Airlangga, Surabaya, Indonesia, unair.ac.id; ^2^ Research Group for Advances in Veterinary Anatomy and Developmental Biology, Faculty of Veterinary Medicine, Universitas Airlangga, Surabaya, Indonesia, unair.ac.id; ^3^ Research Center for Vaccine Technology and Development, Institute of Tropical Disease, Universitas Airlangga, Surabaya, Indonesia, unair.ac.id; ^4^ Department of Health Sciences, Faculty of Vocational Studies, Universitas Airlangga, Surabaya, Indonesia, unair.ac.id; ^5^ Veterinary Stem Cell and Bioengineering Innovation Center, Faculty of Veterinary Science, Chulalongkorn University, Bangkok, Thailand, chula.ac.th

**Keywords:** animals, comparative analysis, regenerative medicine, stem cell characterization, tissue-harvesting site

## Abstract

Adipose tissue possesses great potential for regenerative therapy, making it an important source of mesenchymal stem cells (MSCs). However, studies regarding the characteristics of rabbit adipose‐derived MSCs (Rab‐ADMSCs) obtained from various anatomical areas remain limited. This study aims to investigate the influence of different anatomical sites of adipose tissue on the characteristics, behavior, and differentiation capacity of Rab‐ADMSCs. Three two‐month‐old male White New Zealand rabbits served as donors. Rab‐ADMSCs were obtained from two different anatomical sites, namely, the mesenteric and interscapular areas. The cells were characterized according to their morphology, proliferation rate, clonogenicity, tri‐lineage differentiation capacity, and MSC surface marker expression. The results of this study revealed no morphological differences between Rab‐ADMSCs from two sites. In contrast, variations were noted in several characteristics including cellular behavior, clonogenicity, and tri‐lineage differentiation potential. Phenotypic evaluation on MSC surface markers revealed differences in the expression of CD29 and 49f. In conclusion, the different anatomical sites may influence the characteristics and behavior of Rab‐ADMSCs.

## 1. Introduction

Adipose tissue possesses great potential for both cell‐based and noncell‐based therapies, making it an important source of mesenchymal stem cells (MSCs) [1, 2]. Among the many benefits of this tissue are its high availability, ease of access, and noninvasive sampling procedure [3, 4]. Additionally, this tissue is widely distributed throughout the body and may be extracted from various anatomical sites, allowing the collection of a large number of cells in a single process without the need for complicated expansion procedures [5]. Therefore, the standardization of isolation protocols and characterization of stem cells from various sources are crucial since stem cell‐based therapies are promising in the fields of veterinary surgery and regenerative medicine [6].

To date, several studies investigating the characteristics and differentiation capacity of stem cells, especially MSCs obtained from animals have faced several obstacles. Previous studies reported that the ability of human and canine MSCs to transdifferentiate towards the endodermal lineage has distinct patterns and necessitates protocol modifications [7], which make standardization in biomedical and translational research difficult [8]. A similar issue was also found in adipose tissue‐derived MSCs (ADMSCs) obtained from different anatomical locations. Previous studies have shown that MSCs have differences in morphology, proliferation rate, gene expression, differentiation potential, and surface marker expression in various species such as humans [9], dogs, cats, horses [10], rabbits [11], and rats [12],s suggesting that the characteristics of MSCs depend on species and anatomical site from which the cells are harvested.

Due to their similar phenotype and genotype to humans, rabbits have been widely used as animal models in both human and veterinary medicine. Additionally, rabbit ADMSCs (Rab‐ADMSCs) have been reported to share similarities with human MSCs in terms of cellular and tissue physiology [3, 13, 14]. In contrast, our previous studies on MSC surface markers in Rab‐ADMSCs revealed certain discrepancies compared to human MSCs. Common MSC surface markers such as CD90, CD105, CD44, and CD73 were weakly expressed in rabbit MSCs [3, 15]. Therefore, further exploration to identify and standardize MSC surface markers in rabbits is necessary. Rabbit ADMSCs are known as one of the most potent sources of MSCs. Despite their potential, benefits, and broad applications in both biomedical and translational research for human and veterinary purposes, studies regarding the characteristics of Rab‐ADMSC obtained from various anatomical areas remain limited. This study aims to investigate the influence of different anatomical sites of adipose tissue on the characteristics and differentiation capacity of Rab‐ADMSCs. The findings of this study are expected to contribute to the acceleration of translational studies utilizing rabbit MSCs in both human and veterinary medicine.

## 2. Materials and Methods

### 2.1. Cell Culture

Adipose tissue samples were obtained from the subcutaneous and mesenteric depots of three healthy male White New Zealand rabbits (*n* = 3) with an average body weight of approximately 2 kg. Subcutaneous adipose tissue was collected from the interscapular area, while the mesenteric adipose tissue was collected from the abdominal area. Tissue processing and cell culture were performed according to the protocols described in our previous study [3]. Cells at passages 3–5 were used for all experiments.

### 2.2. Cell Proliferation Assay

A total of 5 × 10^3^ cells were seeded in 96‐well plates (NEST, Jiangsu, China) and incubated for 24, 48, 72, and 96 h to evaluate the proliferation rate. The cell viability was determined using 3‐(4,5‐dimethylthiazol‐2‐yl)‐2,5‐diphenyltetrazolium bromide (MTT) assay. In brief, at each time point, the medium was removed and washed with sterile phosphate‐buffered saline (PBS; Sigma‐Aldrich, Darmstadt, Germany). The cells were incubated for 30 min with 100 μL of MTT solution (0.5 mg/mL in PBS) (Sigma‐Aldrich, Darmstadt, Germany). After the incubation, the solution was removed, followed by washing with PBS. To elute the formazan, 100 μL of dimethyl sulfoxide (DMSO; Sigma‐Aldrich, Darmstadt, Germany) was added. The absorbance of each well was measured using a plate reader (BIOBASE, Shandong, China) at a 570 nm wavelength.

### 2.3. Population Doubling Time (PDT)

The cells were cultured in 24‐well plates (NEST, Jiangsu, China). The initial and final cell densities were counted and recorded to determine the PDT at passages 3 to 5 using Roth’s algorithm [15].

### 2.4. Clonogenic Assay

To determine the clonogenicity of Rab‐ADMSCs, clonogenic assays were conducted. The cells were seeded at a density of 1 × 10^3^ in six‐well plates (NEST, Jiangsu, China) using complete media containing alpha minimum essential medium (α‐MEM) (Gibco, Paisley, UK) supplemented with 10% fetal bovine serum (FBS) (Gibco, New York, USA), 1% penicillin‐streptomycin (Gibco, New York, USA), and 1% amphotericin B (Gibco, New York, USA) for 21 days. The media were replaced every 48 h. The cells were incubated at 37^o^C in a humidified environment with 5% CO_2_. Subsequently, the colonies were stained with 0.1% crystal violet (Sigma‐Aldrich, Darmstadt, Germany) for 30 min at room temperature.

### 2.5. Surface Marker Expression

The MSC surface markers were evaluated using an Attune CytPix Flow Cytometer (Invitrogen, USA) against specific antibodies: CD81, CD49f, CD29, CD9, CD34, and CD45 (Table 1). Mesenteric and interscapular Rab‐ADMSCs at passages 3 and 5 were harvested and resuspended in PBS at a concentration of 1 × 10^6^ cells/mL. All staining procedures were carried out in accordance with the manufacturer’s instructions.

**TABLE 1:** List of primers.

Gene	Accession number	Forward primer reverse primer	Tm (^o^C)	Length (bp)
NANOG	XM_002712762.1	5’–TCA​GCC​TTC​AGC​AGA​TGC​AAG​A–3′	62.26	185
		5’–AGC​AGG​GTA​GAA​GCC​TGG​GTA–3′	62.39	

SOX2	XM_008266557.3	5’–GAC​AGC​TAC​GCG​CAC​ATG​AA–3′	61.35	200
		5’–CTG​TAG​GTG​GGA​GAG​CCG​TT–3′	61.26	

CDKN1A	XM_051854805.1	5’–TTG​GGC​GTG​GAG​ATA​AGG​TGG–3′	62.13	140
		5’–GTC​CGA​CGG​CTG​CGA​CAT–3′	62.80	

TP53	NM_001082404.1	5’–TGA​CAC​GCT​CTC​CTG​AGG​ACT–3′	62.61	70
		5’–CGA​GGC​TGA​GAT​CCG​ACT​GC–3′	62.68	

LPL	NM_001177330.1	5’–CGA​CTG​GGA​ACG​TGT​GTG​TA–3′	59.97	139
		5’–CCA​CAC​ACA​ACC​CTC​TCT​CC–3′	59.96	

PPARγ	XM_051851047.1	5’–CAA​GTA​CGG​CGT​CCA​TGA​GA–3′	59.83	94
		5’–CGT​CAT​GAA​GCC​TTG​TCC​CT–3′	60.04	

COL2A1	NM_001195671.2	5’–GGA​TAG​ACC​CCA​ACC​AAG​GC–3′	60.11	117
		5’–TCC​ACC​AGT​TCT​TCT​TGG​GC–3′	59.89	

SOX9	XM_051825911.1	5’–GCC​CAG​AAG​AGC​CTC​AAA​GT–3′	59.96	95
		5’–TAA​GAG​AGG​TGG​GGA​GGG​TG–3′	59.96	

RUNX2	XM_051855680.1	5’–CTT​CAA​GGT​GGT​AGC​CCT​CG–3′	60.11	154
		5’–CCG​GCC​CAC​AAA​TCT​CAG​AT–3′	60.11	

BMP2	NM_001082650.1	5’–GGA​AAC​GCC​TCA​AAT​CCA​GC–3′	59.83	106
		5’–TAA​AAG​GCG​TGA​TAC​CCC​GG–3′	59.82	

OPN	XM_051840601.1	5’–AGA​CCC​TCC​CGA​GTA​AGT​CC–3′	60.03	111
		5’–CGG​CAT​CGT​CGG​ATT​CAT​TG–3′	59.77	

GAPDH	NM_001082253.1	5’–AGC​TGG​TCA​TCA​ACG​GGA​AG–3′	60.04	110
		5’–GAA​GAC​GCC​AGT​GGA​TTC​CA–3′	60.04	

### 2.6. Tri‐Lineage Differentiation Assay

To induce adipogenic differentiation, 5 × 10^3^ cells were cultured in 24‐well plates (NEST, Jiangsu, China) for 14 days in α‐MEM (Gibco, Paisley, UK) supplemented with 10% FBS (Gibco, Paisley, UK), 10 μM isobutyl‐methylxanthine (Thermo Scientific, NJ, USA), 100 μM indomethacin (Sigma‐Aldrich, MO, USA), 1 μM dexamethasone (Sigma‐Aldrich, MO, USA), and 10 μg/mL insulin (Sigma‐Aldrich, MO, USA). The intracellular lipid droplets were then stained with Oil Red O (Sigma‐Aldrich, MO, USA) staining, and the expression of the adipogenic‐related markers (PPARγ and LPL) was evaluated by RT‐qPCR.

To induce chondrogenic differentiation, 5 × 10^3^ cells were seeded in 24‐well plates (NEST, Jiangsu, China) and treated for 21 days with a chondrogenic induction medium supplemented with 10% FBS (Gibco), 10 ng/mL transforming growth factor‐beta 3 (TGF‐β3) (Thermo Scientific, NJ, USA), 40 μg/mL L‐proline (Sigma‐Aldrich, MO, USA), 50 μg/mL L‐ascorbic acid‐2‐phosphate (Sigma‐Aldrich, MO, USA), and 100 nM dexamethasone (Sigma‐Aldrich, MO, USA). Alcian Blue (Sigma‐Aldrich, MO, USA) was then used to identify glycosaminoglycan, and RT‐qPCR was employed to determine the expression of the chondrogenic mRNA markers SOX9 and COL21A.

To induce osteogenic differentiation, 3 × 10^4^ cells were seeded in 24‐well plates (NEST, Jiangsu, China) and cultured for 21 days using an osteogenic induction medium containing 50 μg/mL L‐ascorbic‐2‐phosphate (Sigma‐Aldrich, MO, USA), 10 mM β‐glycerophosphate (Sigma‐Aldrich, MO, USA), 100 nM dexamethasone (Sigma‐Aldrich, MO, USA), and MEM (Gibco, Paisley, UK) supplemented with 10% FBS (Gibco, Paisley, UK). Subsequently, extracellular matrix mineralization was stained with 0.2 Alizarin Red (Merck, Darmstadt, Germany). RT‐qPCR was used to assess the gene expression of osteogenic mRNA markers (RUNX2, BMP2, and OPN).

The phenotypic staining using Oil Red O, Alcian Blue, and Alizarin Red was quantified using the ImageJ online software (https://ij.imjoy.io), while the gene expression was quantified according the 2^−(ΔΔCt)^ formula.

### 2.7. RNA Extraction and Gene Expression

The total RNA was extracted using the RNAsimple Total RNA Kit (Tiangen, Beijing, China) and quantified using the Qubit Fluorometer 4 (Thermo Scientific, USA). The complementary DNA (cDNA) was synthesized using the FastKing gDNA Dispelling RT SuperMix (Tiangen, Beijing, China) commercial kit and MiniAmp Plus Thermal Cycler (Thermo Scientific, USA). EvaGreen qPCR Master Mix (Biotium, CA, USA) was used to perform quantitative PCR using QuantStudio 5 Real‐Time PCR (Thermo Fisher, USA). The primer sequences used in this study are listed in Table 2. The relative mRNA expression was normalized to GAPDH as a housekeeping gene according to the 2^−(ΔΔCt)^ formula.

**TABLE 2:** List of antibodies.

Antibody	Clone	Clonality	Isotype	Conjugated	Brand and catalog number
CD9	HI9a	Monoclonal	Mouse IgG1,*κ*	PE	Biolegend (312106)
CD81	M38	Monoclonal	Mouse IgG1	FITC	Invitrogen (A15753)
CD29	P4G11	Monoclonal	Mouse IgG1	FITC	Sigma‐Aldrich (MAB1951F)
CD49f	GoH3	Monoclonal	Rat IgG2a,*κ*	PE	Biolegend (313612)
CD45	H130	Monoclonal	Mouse IgG1,*κ*	FITC	Biolegend (304006)
CD34	581	Monoclonal	Mouse IgG1,*κ*	FITC	Biolegend (343504)
Mouse IgG1,*κ*	MOPC‐21	Monoclonal	—	PE	Biolegend (400112)
Mouse IgG1,*κ*	MOPC‐21	Monoclonal	—	FITC	Biolegend (400110)
Rat IgG2a,*κ*	RTK2758	Monoclonal	—	PE	Biolegend (400508)

### 2.8. Statistical Analysis

The results of this study are presented as bar charts using GraphPad Prism Version 9.0 for MacOS (GraphPad Software Inc., California, USA). Nonparametric statistical analysis was used to analyze all experimental data in this study. To compare two independent groups, the Mann–Whitney *U* test was employed, while the Kruskal–Wallis test and pairwise comparison were used to make three or more group comparisons. A *p* value of less than 0.05 was considered significant. The Statistical Package for Social Sciences (SPSS) Statistics version 26 (IBM Corp., NY, USA) was used to analyze all data in this study.

## 3. Results

### 3.1. Influence of Anatomical Sites on Cell Morphology, Pluripotency, Growth Kinetics, Senescence Markers, and Clonogenicity

The results of this study revealed comprehensive data regarding several characteristics of Rab‐ADMSCs isolated from different anatomical sites, both at the phenotypic and molecular levels. The morphology of Rab‐ADMSCs isolated from the mesenteric and interscapular regions shared similar characteristics, such as being fibroblastic and adhering to the bottom of the container (Figure 1(a)). The proliferation capacity of both mesenteric and interscapular Rab‐ADMSCs showed similar trends, indicating that different anatomical sites did not influence the proliferation potential of Rab‐ADMSCs (Figure 1(b)). The PDT analysis revealed an intriguing finding, where the interscapular Rab‐ADMSCs increased almost three times at passage five in comparison to the previous passage. The mesenteric Rab‐ADMSCs, on the other hand, exhibited a comparatively steady PDT from passage 3 to passage 5 (Figure 1(c)). At the molecular level, the expression of pluripotency genes showed a similar pattern. The mRNA expression of NANOG, REX1, and SOX1 in the interscapular Rab‐ADMSCs was higher compared to that of mesenteric Rab‐ADMSCs, although no significant differences in NES mRNA expression were observed (Figure 1(d)). A similar result was seen in the evaluation of the mRNA expression of senescence‐related markers CDKN1A and TP53. Interscapular Rab‐ADMSCs showed higher mRNA expression of CDKN1A and TP53 compared to mesenteric Rab‐ADMSCs (Figure 1(e)). A clonogenicity assay was performed to evaluate the cell proliferation. The results of this study indicated no significant difference in the clonogenic ability of both mesenteric and interscapular Rab‐ADMSCs (Figures 2(a) and 2(b)).

FIGURE 1Evaluation of the morphology and behavior of Rab‐ADMSCs. Rabbit ADMSCs from two different anatomical sites obtained from mesenteric and interscapular depots were evaluated under an inverted microscope (40x magnification and 100x magnification (insert)). Both mesenteric and interscapular Rab‐ADMSCs shared similar morphology (a). In addition, the cell behavior was also evaluated in terms of their proliferation rate (b) and population doubling time (PDT) (c). Gene expression analysis revealed that interscapular Rab‐ADMSCs expressed higher pluripotency markers (d) and senescence‐related markers (e). The Mann–Whitney *U* test was employed to compare two independent groups, while the Kruskal–Wallis test and pairwise comparison were used for three or more group comparisons (*n* = 3). The asterisk indicates a significant difference (*p* < 0.05).

**Figure:**
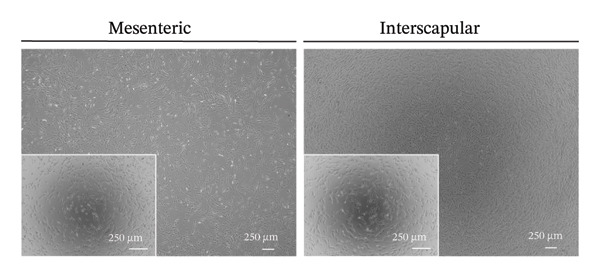
(a)

**Figure:**
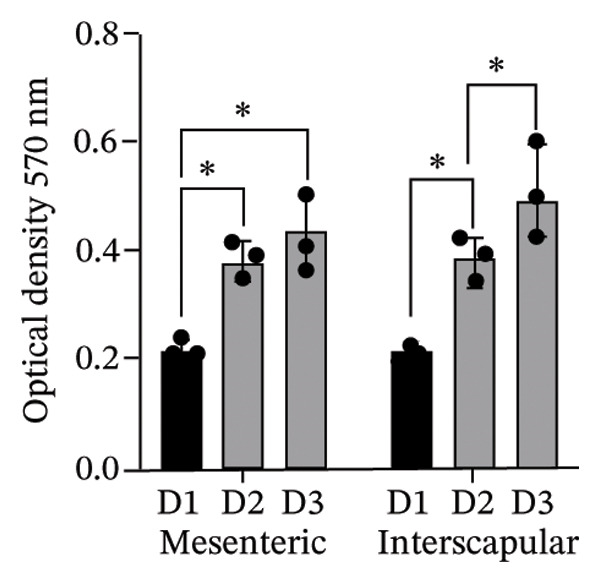
(b)

**Figure:**
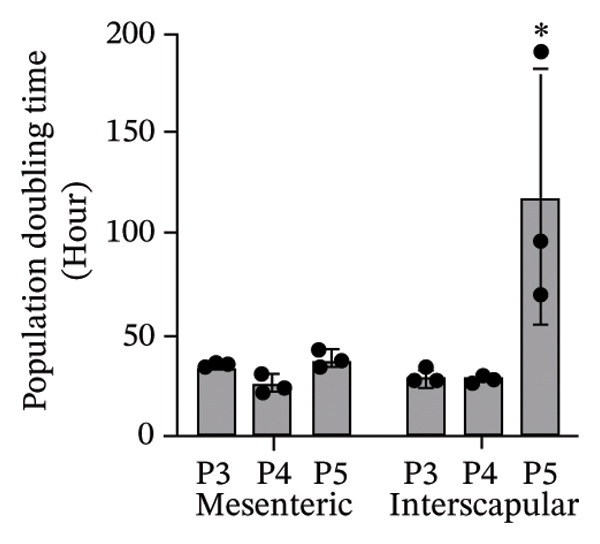
(c)

**Figure:**
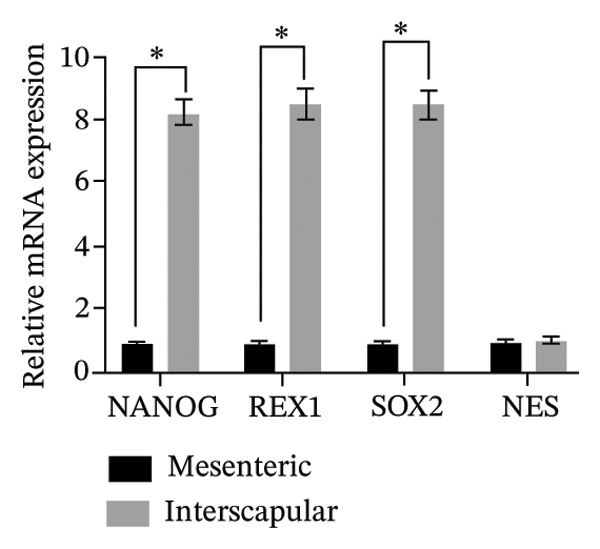
(d)

**Figure:**
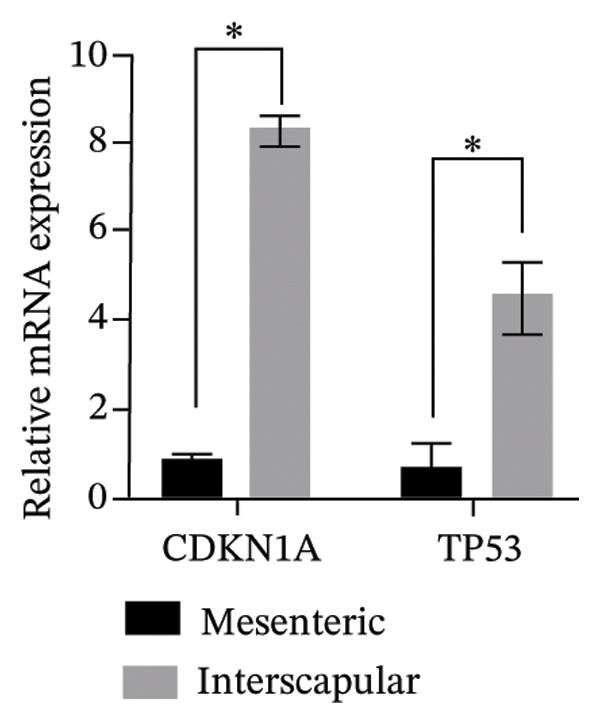
(e)

FIGURE 2Clonogenicity assay of Rab‐ADMSCs. To determine the cell clonogenicity, both Rab‐ADMSCs were stained using crystal violet and evaluated under a microscope (a). Mesenteric and interscapular Rab‐ADMSCs shared a similar number of colonies (b), suggesting that the anatomical site did not influence the clonogenicity of Rab‐ADMSCs. A nonparametric (Mann–Whitney *U*) test was used to assess the differentiation between two groups (*n* = 3). The asterisk indicates a significant difference (*p* < 0.05).

**Figure:**
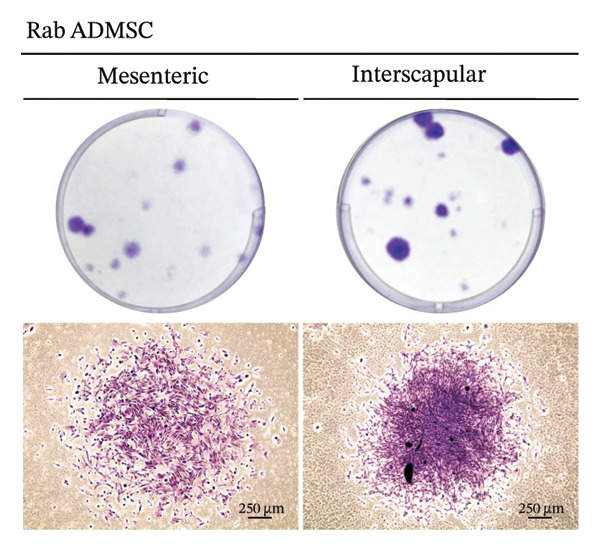
(a)

**Figure:**
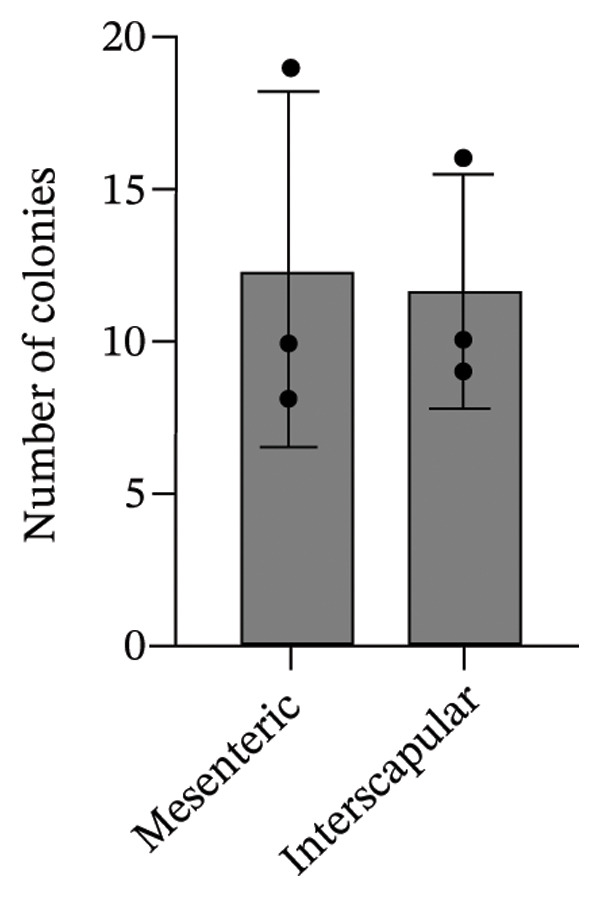
(b)

### 3.2. Tri‐Lineage Differentiation Capacity

The differentiation capacity of Rab‐ADMSCs into osteocytes, chondrocytes, and adipocytes was evaluated phenotypically under the microscope. Rab‐ADMSCs were cultured in three different induction media: osteogenic, adipogenic, and chondrogenic. The results of this study showed that the two different anatomical locations did not influence the differentiation capacity of mesenteric and interscapular Rab‐ADMSCs, as illustrated in Figure 3(a). The phenotypic evaluation using histochemical staining with Alizarin Red, Alcian Blue, and Oil Red O indicated that both Rab‐ADMSCs were capable of differentiating into osteocytes, chondrocytes, and adipocytes, as demonstrated by the red, blue, and red colors, respectively. Furthermore, the staining was quantified using ImageJ. The results revealed that the percentage area of Oil Red O and Alcian Blue staining were significantly higher in mesenteric Rab‐ADMSCs, indicating their capacity for adipogenic and chondrogenic differentiation. In contrast, both Rab‐ADMSCs showed comparable osteogenic differentiation capacity, although a trend toward the higher capacity was observed in interscapular Rab‐ADMSCs (Figures 3(b) and 3(c)). The results of gene expression analysis at the mRNA level using RT‐qPCR showed that osteogenic marker genes (RUNX2, BMP2, and OPN), chondrogenic marker genes (COL2A1 and SOX9), and adipogenic marker genes (LPL and PPARγ) were all expressed in both induced Rab‐ADMSCs (Figures 4(a), 4(b), 4(c)). In osteogenic differentiation, the expression of the BMP2 gene in interscapular Rab‐ADMSCs appeared higher compared to mesenteric Rab‐ADMSCs, but not in the expression of the RUNX2 and OPN genes (Figure 4(a)). An interesting observation was seen in adipogenic differentiation, where the expression of the LPL gene in interscapular Rab‐ADMSCs appeared higher compared to mesenteric Rab‐ADMSCs, whereas the expression of the PPARγ gene was higher in mesenteric Rab‐ADMSCs (Figure 4(b)). The expression of the SOX9 and COL2A1 genes in chondrogenic differentiation also showed a similar pattern. Interscapular Rab‐ADMSCs expressed the SOX9 gene at higher levels compared to mesenteric ADMSCs, whereas the expression of the COL2A1 gene was lower (Figure 4(c)).

FIGURE 3Phenotypic evaluation of tri‐lineage differentiation staining. The adipogenic lineage was stained with oil red O, osteogenic with alizarin red, and chondrogenic with alcian blue (a). The quantification of phenotypical staining using ImageJ online software revealed the dynamics of tri‐lineage differentiation capacity into adipocytes, osteocytes, and chondrocytes (3b–d). A nonparametric (Mann–Whitney *U*) test was used to assess the differentiation between two groups (*n* = 3). The asterisk indicates a significant difference (*p* < 0.05).

**Figure:**
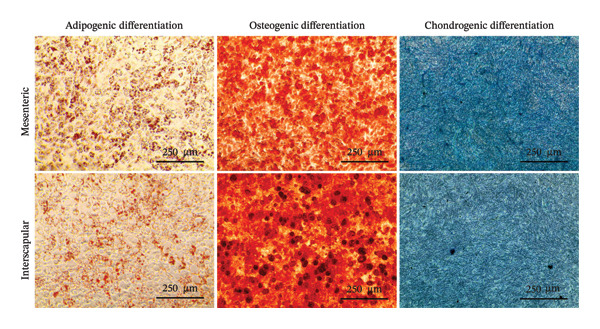
(a)

**Figure:**
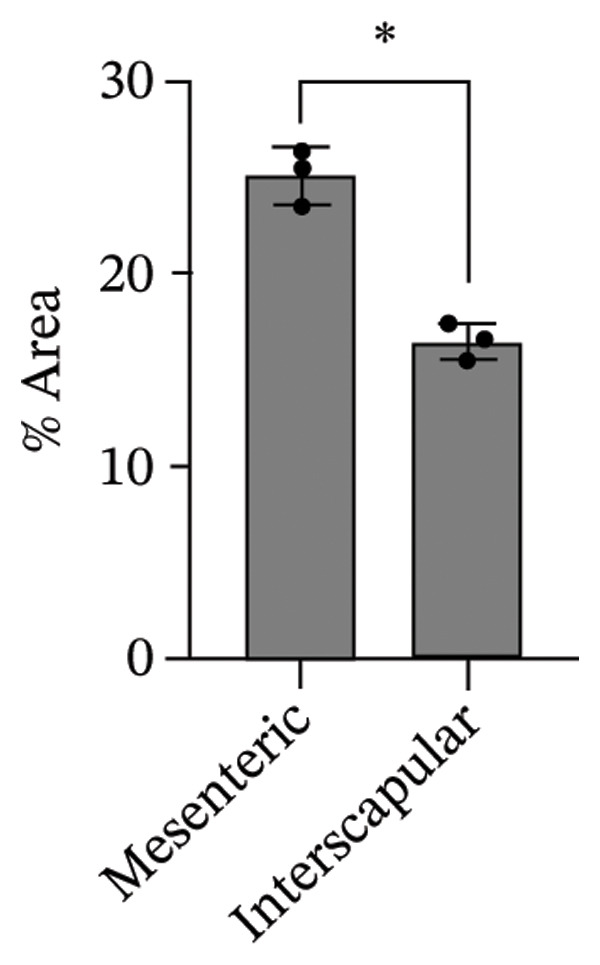
(b)

**Figure:**
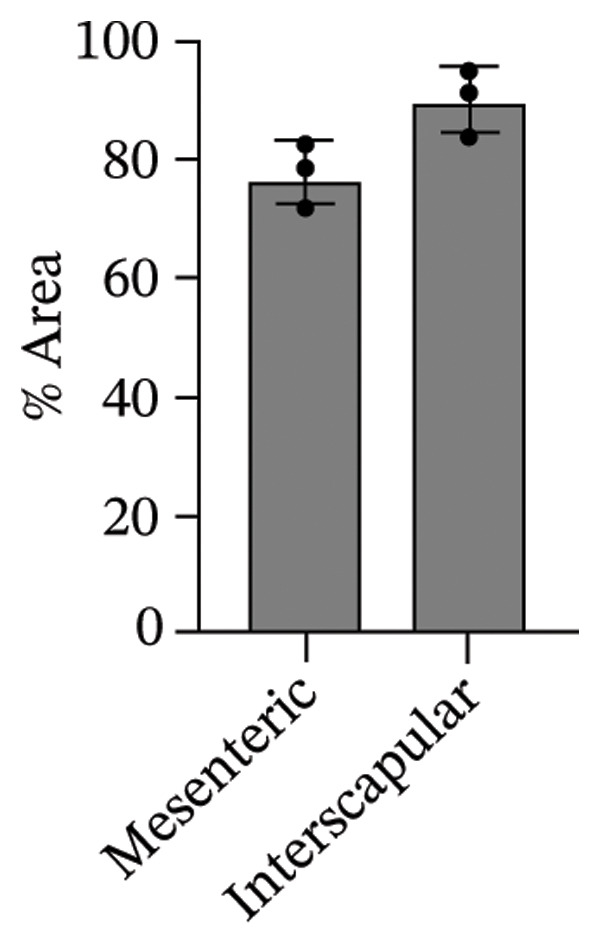
(c)

**Figure:**
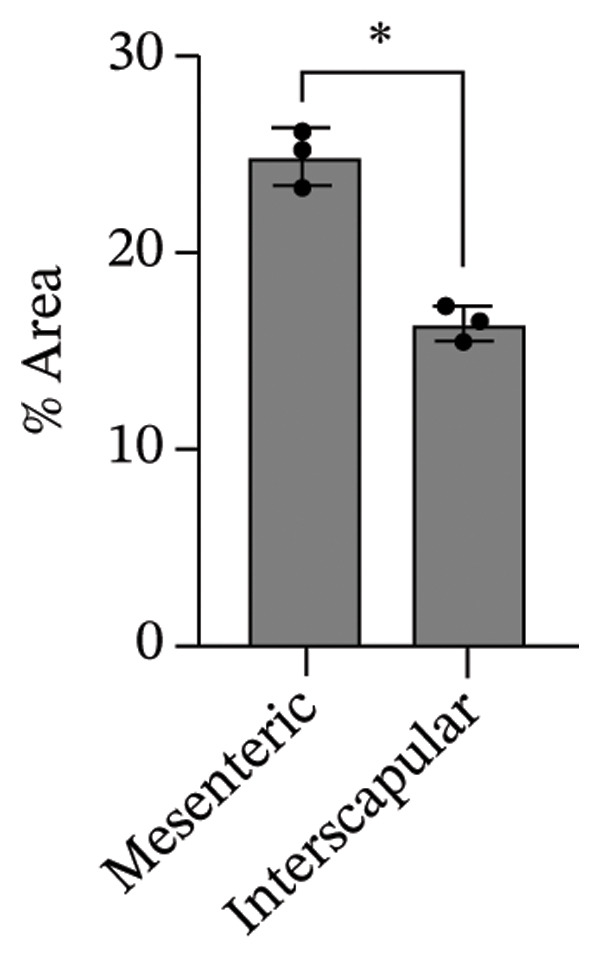
(d)

FIGURE 4Gene expression analysis of the tri‐lineage differentiation. Osteogenic‐related genes (RUNX2, BMP2, and OPN) (a), adipogenic‐related genes (LPL and PPARγ) (b), and chondrogenic‐related genes (COL2A1 and SOX9) (c) were expressed in both mesenteric and interscapular Rab‐ADMSCs. The Kruskal–Wallis test and pairwise comparison were used for three or more group comparisons (*n* = 3). The asterisk indicates a significant difference (*p* < 0.05).

**Figure:**
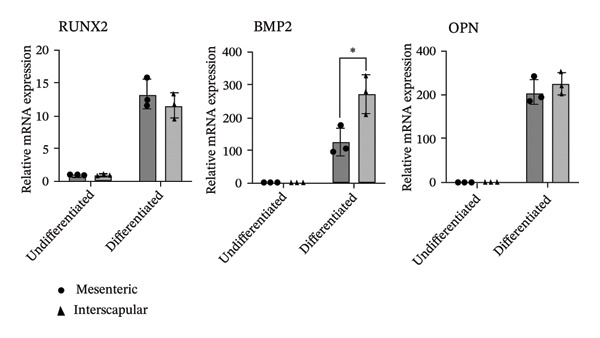
(a)

**Figure:**
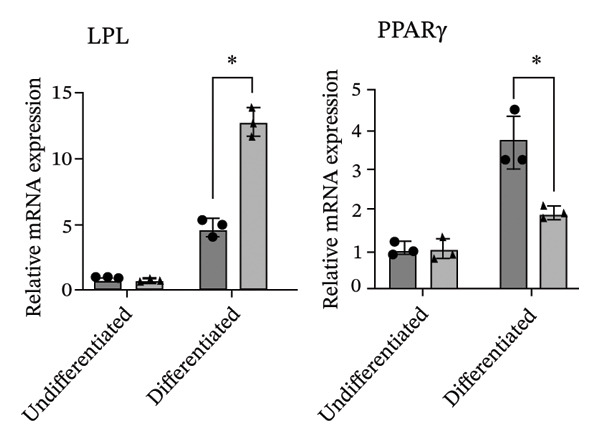
(b)

**Figure:**
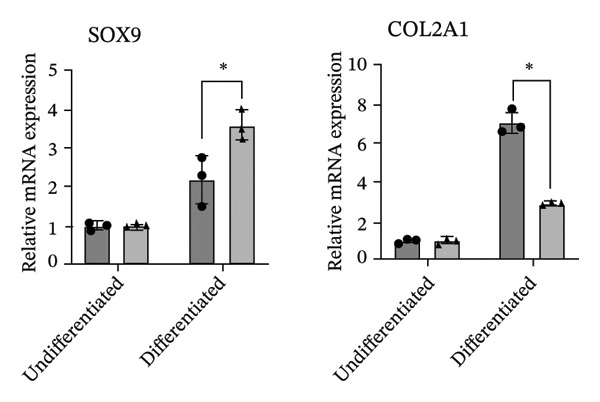
(c)

### 3.3. Phenotypic Characterization of Rab‐ADMSCs

Phenotypic analysis using flow cytometry was performed to determine the expression of specific surface protein markers for MSCs. The results showed varying results in both Rab‐ADMSCs (Figure 5(a)). Specific markers CD81 and CD9 were highly expressed in both Rab‐ADMSCs, whereas the expression of CD49f and CD29 in both Rab‐ADMSCs showed varying results. The expression of the CD49f surface marker was higher in the mesenteric Rab‐ADMSCs, whereas the expression of the CD29 marker was lower. The expression of CD49f in the interscapular Rab‐ADMSCs appeared lower, but the expression of CD29 was higher compared to the mesenteric Rab‐ADMSCs (Figure 5(b)). Both hematopoietic stem cell (HSC) markers, CD34 and CD45, were not expressed in both Rab‐ADMSCs.

FIGURE 5Phenotypic evaluation of MSC surface markers (passage 5). Both interscapular and mesenteric Rab‐ADMSCs were phenotypically characterized using flow cytometry (a). According to the evaluation, the expression of CD81 and CD9 was consistently high in both Rab‐ADMSCs, while CD49f and CD29 showed varied expressions from low to moderate. Both Rab‐ADMSCs did not express hematopoietic stem cell surface markers, namely, CD34 and CD45 (b). The gating strategy was performed according to the live single cells. A nonparametric (Mann–Whitney *U*) test was used to assess the differentiation between two groups (*n* = 3). The asterisk indicates a significant difference (*p* < 0.05).

**Figure:**
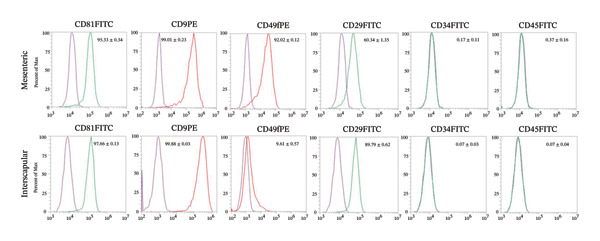
(a)

**Figure:**
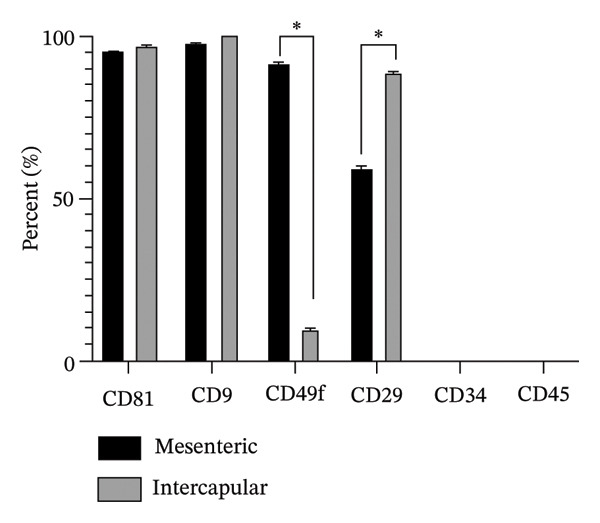
(b)

## 4. Discussion

This comprehensive study examined the potential influence of diverse anatomical locations on the morphology, proliferation, differentiation capacity, and surface marker expression of Rab‐ADMSCs. A number of studies have attempted to look at the characteristics of Rab‐ADMSCs, but information on discrepancies arising from various anatomical sites remains limited [11, 14]. Nonetheless, a number of investigations have documented differences in MSCs derived from various anatomical sites in humans, rats, dogs, cats, and horses [10, 12]. In this study, microscopical observations of Rab‐ADMSCs revealed that cells isolated from the mesenteric and interscapular regions shared similar characteristics such as adherence and spindle‐like shape. These results are consistent with previous investigations that reported the morphological assessment of different MSCs [3, 13, 14, 16]. At passages 3 and 4, the growth kinetic of Rab‐ADMSCs derived from the mesenteric and interscapular regions exhibited similar patterns. However, in contrast to mesenteric MSCs, the PDT of interscapular Rab‐ADMSCs increased threefold at passage 5. This behavior may be attributed to the elevated expression of the senescence mRNA markers CDKN1A and TP53 in the interscapular Rab‐ADMSCs. Previous studies have reported that senescence can cause proliferation arrest [17] and reduce growth rate [18]. Through the interaction between p53/TP53 and p21/CDNKN1A, which results in cell cycle arrest, the induction of cellular senescence by activating the p53 family contributes to tumor suppression and accelerated aging. However, the p53 family also promotes longevity and tissue homeostasis by safeguarding renewable stem cells [19, 20]. Furthermore, transcription factors such as REX1, SOX2, and NANOG have an important role in regulating self‐renewal and differentiation in stem cells [12], while NES is required for stem cell survival, self‐renewal, and proliferation, as well as regulating cell differentiation and migration [21]. In this study, pluripotent mRNA markers of REX1, SOX2, and NANOG were expressed higher in interscapular Rab‐ADMSCs compared to mesenteric Rab‐ADMSCs, although the mRNA expression of NES was similar. These findings indicate the presence of the transcription factors regulating pluripotency in both Rab‐ADMSCs, consistent with the clonogenicity analysis which revealed that both mesenteric and interscapular Rab‐ADMSCs had a similar number of colonies. In other words, this finding is consistent with a previous study on clonogenicity [13].

Tri‐lineage differentiation into osteocytes, adipocytes, and chondrocytes is a major defining characteristic of MSCs. According to previous studies, the phenotypic feature of positive histochemical specific staining (Oil Red O, Alizarin Red, and Alcian Blue) and the elevation of the expression of the lineage‐associated genes show that mesenteric and interscapular Rab‐ADMSCs have tri‐lineage differentiation characteristics [3, 13, 16, 22]. The findings of this study demonstrated that, in response to in vitro induction media in adipogenic, osteogenic, and chondrogenic differentiation, both mesenteric and interscapular Rab‐ADMSCs were able to change their phenotypes by forming proteoglycan, calcium deposits, and lipid droplets, respectively. The mRNA expression of lineage‐associated markers is also consistent with an earlier study [3]. According to the phenotypic evaluation through specific histochemical staining in this study, all of the positive results were notable in both mesenteric and interscapular Rab‐ADMSCs. Although the quantification analysis in differentiation phenotypic evaluation was not reported and may be considered a limitation, the comprehensive analysis on the dynamics of the gene expression using RT‐qPCR was described in this study. At the molecular level, the osteogenic differentiation revealed that the expression of BMP2 was significantly higher in interscapular Rab‐ADMSCs, while the mRNA levels of RUNX2 and OPN were comparable. Important key players in the osteogenic differentiation include OPN, BMP2, and RUNX2. RUNX2 is a crucial transcription factor that regulates the expression of osteogenic genes, while the synthesis of OPN indicates that the cells are progressing into the maturation stage and osteoblastic phenotypes are present in the final stage of differentiation. Additionally, BMP2 functions as an inducer in the intermediate stage, promoting MSC development into osteoblasts [23, 24]. The dynamics of mRNA expression in both Rab‐ADMSCs indicated the variations in osteogenic differentiation. Although the mRNA expression of OPN as a late‐stage marker of osteogenic differentiation was not significantly different, the interscapular Rab‐ADMSCs showed trend‐level of earlier maturation. Similar results were noted in the levels of RUNX2 mRNA as early‐stage markers, where the interscapular Rab‐ADMSCs tended to have lower levels. These dynamics suggest that the interscapular Rab‐ADMSCs may differentiate into a mature stage earlier than the mesenteric Rab‐ADMSCs. Higher levels of pluripotency‐associated gene expression may correlate with higher BMP2 expression in the interscapular Rab‐ADMSCs. This finding is consistent with a previous study on human MSCs which found a positive correlation between elevated BMP2 expression and higher expression of pluripotency‐associated genes [25]. The maturation of preadipocytes into adipocytes requires the induction of LPL and PPARγ mRNA [26]. A similar study reported that the PPARγ mRNA expression was elevated on days 14 and 21 after the induction, which might contribute to adipocyte maturation [27]. On the other hand, the expression of LPL which plays as a crucial indicator of adipocyte maturation, is positively correlated with the activation of PPARγ as a key regulator of adipogenesis. LPL expression is more noticeable in the latter stages of differentiation, indicating the developmental progression of adipocytes and their capacity for lipid storage, whereas PPARγ expression tends to elevate early [28, 29]. The findings of this study indicated that the interscapular Rab‐ADMSCs tend to differentiate into mature adipocytes earlier than the mesenteric Rab‐ADMSCs. In chondrogenic differentiation, the transcription factor SOX9 is essential to trigger the production of Type II collagen mRNA, COL2A1. As a key transcription factor in chondrogenesis, SOX9 expression depends on its ability to bind to particular areas of the COL2A1 enhancer, namely, the 48‐bp chondrocyte‐specific enhancer. This binding is a crucial step in the development of cartilage, which involves proper regulation of COL2A1 (collagen Type II) expression. High COL2A1 expression is typically associated with high SOX9 mRNA expression, especially in chondrocytes [30]. Higher mRNA expression of COL2A1 indicates that mesenteric Rab‐ADMSCs undergo maturation earlier compared to interscapular Rab‐ADMSCs, suggesting that the discrepancy in chondrogenic differentiation in rabbit MSCs is notable in this study. Similar results regarding the differences in the mRNA expression of COL2A1 and SOX9 were also reported by a previous study [31]. This present study revealed that the anatomical origin of ADMSCs may influence this discrepancy, which is also consistent with a prior study which reported that a distinct expression may be influenced by the anatomical sites and lineage imprinting [12].

The phenotypic evaluation of rabbit MSCs remains challenging. The limitation of commercially available antibodies as required by the International Society for Cell Therapy (ISCT) is one major obstacle for the characterization of rabbit MSCs. The previous study revealed that CD90, CD105, and CD73 were weakly expressed, whereas CD44 was moderately expressed [3]. Therefore, finding new potential surface markers for rabbit MSCs is necessary. In this study, phenotypic evaluation of MSC surface markers using flow cytometry revealed variations in the expression of particular surface markers between interscapular and mesenteric Rab‐ADMSCs. Positive markers from earlier studies, such as CD9, CD29, CD49f, and CD81, and negative markers such as CD45 and CD34 were included in this study [3]. The results of this study showed that CD9 and CD81 were highly expressed, while hematopoietic surface markers such as CD45 and CD34 were negatively expressed. These findings are consistent with earlier findings on Rab‐ADMSCs isolated from subcutaneous and mesenteric adipose tissue [3, 14, 22, 32]. In contrast, there was a notable variation in CD29 expression, which was lower in mesenteric Rab‐ADMSCs and moderate in interscapular Rab‐ADMSCs. These findings are consistent with several previous studies reporting high to moderate expression of CD29 [3, 16, 33, 34]. A notable variation was also seen in the expression of CD49f that was lower in the interscapular Rab‐ADMSCs compared to the mesenteric Rab‐ADMSCs. The expression CD49f is correlated with the expression of genes related to pluripotency, such as OCT4 and SOX2, which are known to bind to the promoters of integrin *α*6 (CD49f) that support transcription and are associated with cell pluripotency [16]. Low expression of CD49f is correlated with higher PDT in interscapular sites. Although the cells exhibited a higher expression of SOX2, the results of this study revealed that higher expression of the senescence markers CDKN1A and TP53 may contribute to the variations in the expression of this surface marker. Despite the novel findings on the different characteristics of both mesenteric and interscapular Rab‐ADMSCs, the small sample size used in this study might be a limitation. The utilization of small number of animals was based on the ethical consideration related to the 3R (replacement, reduction, and refinement) principles. In addition, this study serves as an initial study to investigate the anatomical sites as MSC sources and their characteristics, both phenotypically and genotypically, to explore a new concept and establish the methodology for further research.

## 5. Conclusion

This study reveals differences in the growth kinetics, pluripotency, senescence, and clonogenicity of Rab‐ADMSCs obtained from different anatomical sites. In addition, comprehensive molecular analyses on tri‐lineage differentiation of Rab‐ADMSCs show that the interscapular Rab‐ADMSCs tend to differentiate into mature osteocytes and adipocytes earlier than the mesenteric Rab‐ADMSCs. Meanwhile, in chondrogenic maturation, the mesenteric Rab‐ADMSCs tend to differentiate earlier. Phenotypic evaluation regarding the MSC surface markers shows that the expression of CD81 and CD9 is consistently higher in both mesenteric and interscapular Rab‐ADMSCs. In contrast, the expression of CD49f and CD29 in both Rab‐ADMSCs varies from low to moderate. The findings of this study indicate that the different anatomical sites as a source of rabbit ADMSCs may influence the behavior and characteristics of MSCs, suggesting that an appropriate anatomical site should be selected carefully. The results of this study may be beneficial for further research on the utilization of rabbit ADMSCs to accelerate the translational research into clinical settings in both human and veterinary medicine.

## Author Contributions

Suryo Kuncorojakti: writing the manuscript (reviewing and editing), data validation, supervision, methodology, funding acquisition, formal analysis, data curation, and conceptualization; Ahmad Aswin: writing the manuscript (original draft), methodology, investigation, formal analysis, and data curation; Diyantoro: investigation, formal analysis, and data curation; Helen Susilowati: investigation, formal analysis, and data curation; Ira Sari Yudaniayanti: investigation, formal analysis, and data curation; Yeni Dhamayanti: investigation, formal analysis, data curation; Lina Susanti: investigation, formal analysis, and data curation; Watchareewan Rodpraser: data validation, supervision, methodology, data curation, and conceptualization.

## Funding

This study was funded by Universitas Airlangga through Airlangga Research Fund Fiscal year 2025 with a grant number 1801/UN3.LPPM/PT.01.03/2025.

## Ethics Statement

All experimental protocols of this study were approved by the Institutional Animal Care and Use Committee (IACUC) of the Faculty of Veterinary Medicine, Airlangga University with an approval number 1.KEH.112.07.2024.

## Conflicts of Interest

The authors declare no conflicts of interest.

## Data Availability

The data that support the findings of this study are available from the corresponding author upon reasonable request.
